# Delayed bronchial perforation after bulla cauterization with soft coagulation system

**DOI:** 10.1186/s40792-021-01327-z

**Published:** 2021-11-18

**Authors:** Sakiko Kumata, Katsunari Matsuoka, Shinjiro Nagai, Mitsuhiro Ueda, Yoshinori Okada, Yoshihiro Miyamoto

**Affiliations:** 1 Department of Thoracic Surgery, Institute of Development, Aging and Cancer, Tohoku University, 4-1 Seiryomachi, Aobaku, Sendai 980-8575 Japan; 2 Department of Thoracic Surgery, National Hospital Organization Himeji Medical Center, Himeji, Japan

**Keywords:** Soft coagulation system, Thermal injury, Bronchial perforation, Bronchopleural fistula

## Abstract

**Background:**

Soft coagulation is widely used for hemostasis because of its significant advantage in inducing tissue coagulation and denaturation without carbonization. However, a few cases of airway damage have been reported at the site, where soft coagulation was directly applied.

**Case presentation:**

We encountered an unusual case of delayed perforation of the intermediate bronchial trunk observed on 24 days after cauterization of the right S6 bulla adjacent to the bronchus. Chest computed tomography revealed a large fistula between the intermediate bronchial trunk and the cauterized bulla in the right S6. Bronchoscopy showed a large fistula at the membranous portion of the intermediate bronchial trunk. We presumed that the bronchial perforation resulted from thermal damage to the intermediate bronchial trunk during bulla cauterization and the bronchial perforation induced infection in the bulla. Resection of the infectious bulla and the intermediate bronchial trunk, followed by end-to-end bronchial anastomosis and a pedicled intercostal muscle flap coverage, was performed.

**Conclusions:**

The severe airway damage resulting in perforation developed even without direct contact between the electrode tip and the bronchial wall, provoking the need for special attention to the duration of cauterization and location, where it is used.

## Background

Soft coagulation system (SCS), (VIO3, ERBE Elektromedizin GmbH, Germany) is a technology that induces tissue coagulation and denaturation without carbonization by regulating its output voltage to below 200 V. In the field of thoracic surgery, SCS has been widely used mainly for hemostasis of bleeding from pulmonary, bronchial, and intercostal vessels [1]. In addition, several studies have shown that SCS could be used for bulla cauterization and effective in treating bullas, where resection might not be performed, especially those close to the hilar structure and on the interlobar surface [2, 3]. In contrast, several studies have reported unexpected tracheobronchial ischemia or perforation at the site, where soft coagulation was directly applied during pulmonary resection [4, 5]. Here, we report an unusual case of delayed perforation of the intermediate bronchial trunk (IBT) after bulla cauterization using SCS.

## Case presentation

**Fig. 1:**
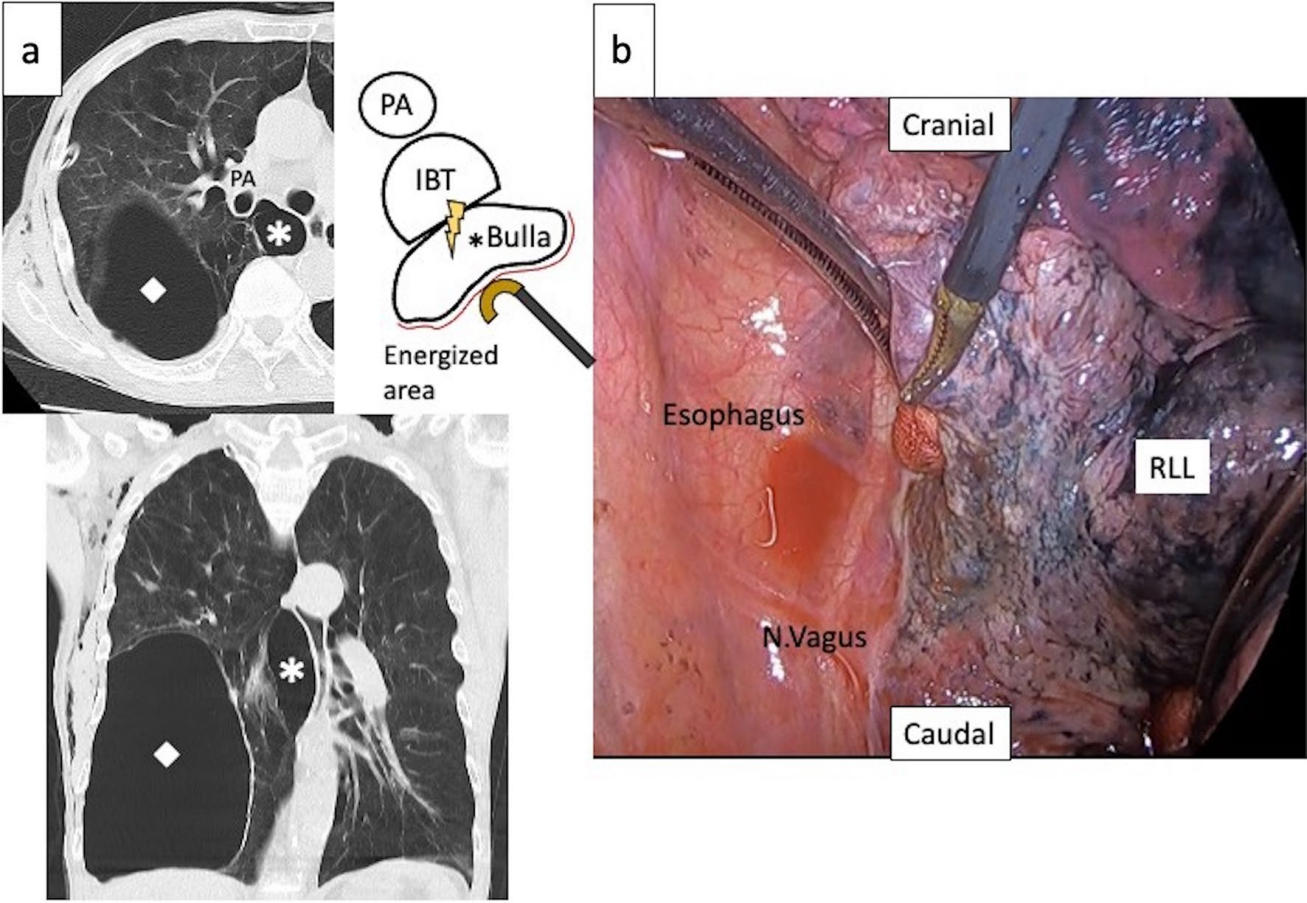
**a** Chest computed tomography shows a large bulla in the right segment 8 (open diamond) and a smaller bulla in segment 6 (*) located in contact with the intermediate bronchial trunk (IBT). A schema represents the anatomical position of the IBT and the energized area of the bulla in the right segment 6. **b** Thoracoscopic review of the bulla cauterization. The giant bulla in the right segment 6 was cauterized using soft coagulation system. *RLL* right lower lobe

**Fig. 2:**
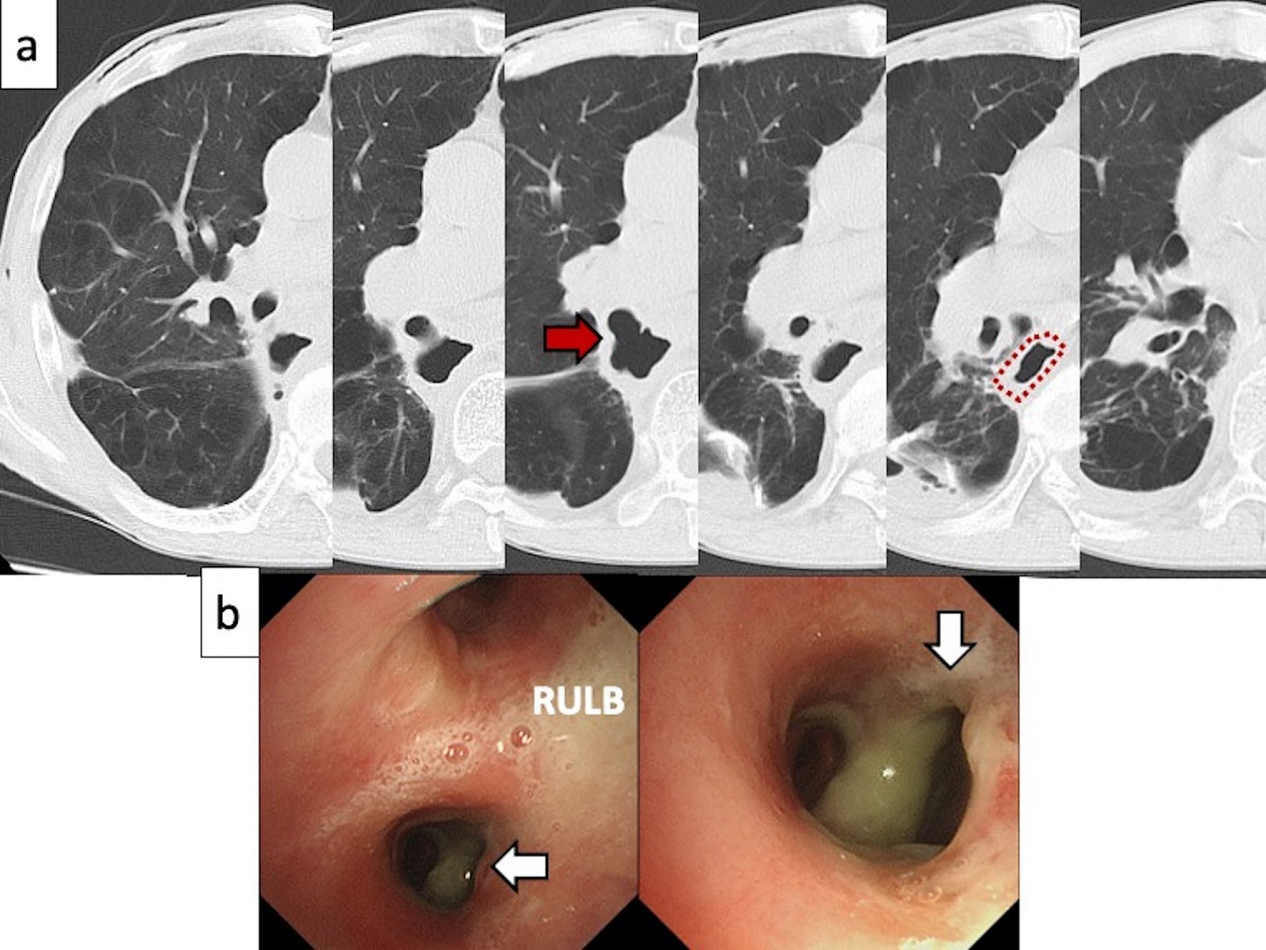
**a** Computed tomography on postoperative day 24 shows a fistula on the IBT (arrow) to the inside of the bulla in the segment 6 of the right lung (dotted line). **b** Bronchoscopic finding reveals a large perforation of the IBT (arrows). *RULB* right upper lobe bronchus

A 69-year-old man with a smoking history of 125 pack-years developed pneumothorax and was referred to our hospital. The patient was suffering from shortness of breath on exertion resulting from chronic obstructive pulmonary disease. Chest computed tomography (CT) on admission demonstrated a large bulla in the right segment 8 (S8) and a smaller bulla in the right S6 (Fig. 1a). Under 3-port thoracoscopic surgery, the bulla of the right S8 was stapled, and that of the right S6, which was located close to the hilar structures and unfeasible for stapling, was cauterized using SCS (Fig. 1b). Cauterization was performed using the dorsal side of thoracoscopic forceps (T1268, Olympus, Japan) set at effect 6 for approximately 50 s in total. The surgery was completed uneventfully; however, the patient underwent pleurodesis on postoperative day (POD) 6 for persistent air leakage. He was discharged from the hospital on POD 12 without oxygen inhalation. On day 24, the patient visited our outpatient clinic complaining of fever and rust-colored sputum. Blood biochemical examination showed an elevated leukocyte of 11,500/mm^3^ and C-reactive protein of 20.5 mg/dL. Chest CT revealed a large fistula between the IBT and cauterized bulla in the right S6 (Fig. 2a). Bronchoscopy showed a large fistula at the membranous portion of the IBT (Fig. 2b). After reviewing the preoperative CT (Fig. 1a), we presumed that the bronchial perforation resulted from thermal damage to the IBT during bulla cauterization and induced infection in the bulla. Resection of the infectious bulla and IBT, followed by end-to-end bronchial anastomosis and a pedicled intercostal muscle flap coverage, was performed. The pathological findings of the resected IBT showed that a part of the IBT wall that lacks a membranous portion was necrotic with exudative necrotic debris on the surface. The surrounding area showed fibrosis and granulation tissue formation. The patient was discharged on POD 73 after the second operation.

## Discussion

Two cases of bronchial ischemia and one case of bronchial perforation associated with SCS were first reported by Shibano et al. in 2015 [4]. Severe mucosal ischemia was noted by intra- or postoperative bronchoscopy in the former 2 cases, and a bronchopleural fistula at the lateral wall of the right B4 had developed in the other patient on POD 15 after right lower lobectomy. Hashimoto et al. have reported a case of tracheal perforation following right upper lobectomy and superior mediastinal lymph node dissection, which was noticed on POD 37 [5]. In all 4 cases, the location of ischemia or perforation was exactly the same site, where the ball electrode tip connected to SCS was directly applied for hemostasis; therefore, the authors undoubtedly assumed that soft coagulation caused the bronchial wall damage.

Distinct from previously reported cases, the patient in this case report had bronchial perforation after bulla cauterization, even though the electrode tip was not directly in contact with the bronchial wall. Based on the operative findings and preoperative CT images, we speculated that the IBT was damaged through the giant bulla during cauterization, which required a relatively long time. Tissues with low blood flow, such as the bronchus, are prone to thermal damage because of the lack of blood flow, which acts as a radiator. Compared with normal lung parenchyma and mediastinal tissues, which are rich in moisture content, low-moisture tissue is also estimated to be easily thermally damaged. The unexpected thermal damage to the IBT by relatively low temperatures with SCS was thought that the bulla inside was heated by prolonged soft coagulation, and the heat was directly transferred to the bronchial wall adjacent to the cyst. This finding warns us to take careful precautions against possible severe damage to the bronchial or tracheal wall by SCS, regardless of whether there is direct contact of the electrode tip with the airway. When SCS is inevitably applied to the airway wall or its adjacent tissue for a long time, intraoperative bronchoscopy should be performed.

Moreover, note that 3 reported cases of airway perforation associated with SCS, including the patient in this case reported, occurred on PODs 13, 21, and 37, respectively. Verkindre et al. have chronologically examined the histological changes in large airways after endobronchial application of soft coagulation in animals [6]. They have shown that coagulation necrosis of the mucosa alone and acute inflammation of the bronchial structure in the early phase (48 h) dramatically resulted in transmural fibrosis and cartilage destruction in the late phase (6 weeks). This suggests that tissue damage to the airway wall by SCS remains over several weeks and that the damage caused by SCS, depending on the extent of the damage, is irreversible. Though covering the damaged area with biological tissue, such as muscle or adipose flap, is deemed appropriate to prevent airway perforation, the effect of this technique is unknown.

## Conclusions

In summary, this case report suggests the need for special attention to the possibility of invisible thermal damage by soft coagulation during surgery, regardless of whether there is direct contact of the electrode tip with the tracheobronchial walls. Most importantly, since the changes caused by soft coagulation may be irreversible, depending on the extent of the damage, we must be careful about cauterization duration and extent. Delayed airway perforation should be taken into account as a possible severe complication after thoracic surgery in which soft coagulation is extensively applied.

## Data Availability

Case report data and patient’s consent form are available.

## Abbreviations

SCS: Soft coagulation system
IBT: Intermediate bronchial trunk
CT: Computed tomography
POD: Postoperative day
